# Mammalian target of rapamycin complex 2 regulates inflammatory response to stress

**DOI:** 10.1007/s00011-012-0542-7

**Published:** 2012-08-17

**Authors:** Desmond Mascarenhas, Sheri Routt, Baljit K. Singh

**Affiliations:** 1 Mayflower Organization for Research and Education, 525 Del Rey Avenue, Suite B, Sunnyvale, CA 94085 USA; 2 Piedmont Research Center, Morrisville, NC USA; 3 Protigen Inc, Sunnyvale, CA USA

**Keywords:** Nephrilin, mTORC2, Substance P, NGAL, UCHL1

## Abstract

**Objective and design:**

To explore the role of mammalian target of rapamycin 2 (mTORC2) in the activation of inflammatory and oxidative responses in rodent models of acute injury and metabolic stress.

**Material:**

The impact of nephrilin, an inhibitor of mTORC2 complex, was assessed in three CD-1 mouse models of acute xenobiotic stress and in a hypertensive Dahl rat model of metabolic stress.

**Methods:**

Animals received daily subcutaneous bolus injections of saline or 4 mg/kg nephrilin. Tissues were assayed by ELISA, gene arrays and immunohistochemical staining.

**Results:**

Nephrilin significantly inhibited elevations in plasma tumor necrosis factor-alpha, kidney substance P, and CX3CR1, and urinary lipocalin-2 [urinary neutrophil gelatinase-associated lipocalin (uNGAL)] in models of acute xenobiotic stress. UCHL1 gene expression levels dropped and plasma HMGB1 levels rose in the rhabdomyolysis model. Both effects were reversed by nephrilin. The inhibitor also blocked diet-induced elevations of uNGAL and albumin-creatinine ratio (UACR) as well as kidney tissue phosphorylation of PKC-beta-2-T641 and p66shc-S36, and reduced dark ring-like staining of nuclei by anti-phos-p66shc-S36 antibody in frozen sections of diseased kidneys from hypertensive Dahl rats fed an 8 % NaCl diet for 4 weeks.

**Conclusions:**

Taken together, our results suggest a role for mTORC2 in the inflammatory-oxidative responses to stress.

## Introduction

Under stress, mammals unleash inflammatory cytokines and cellular reactive oxygen species via linked mechanisms that are only partially understood [1, 2]. In humans, systemic inflammatory responses to traumatic stress can lead to sepsis and high mortality in the intensive care unit, especially when marked by kidney injury [3]. Urinary neutrophil gelatinase-associated lipocalin (uNGAL) has been used as a reliable early marker of such injury in critical care settings [4, 5]. The inflammatory and oxidative processes underlying the pathology of these life-threatening conditions are not well characterized, but are hallmarks of a variety of insults including ischemic injury, rhabdomyolysis, burns, and xenobiotic and physical trauma [6]. A parallel phenomenon of underlying oxidative damage triggered by stress has also been suggested for disease conditions ranging from diabetes to cancer [7, 8]. We are interested in investigating conserved mechanisms that may link stress to dysfunctional inflammation and oxidative circuitry.

Rictor-containing mammalian target of rapamycin 2 (mTORC2) is an evolutionarily conserved regulatory complex. Unlike its well-known counterpart, Raptor-containing mTORC1, mTORC2 is rapamycin-insensitive and believed to play a role in cell shape, cell motility, and responses to stress [9, 10] by phosphorylating AGC family kinases such as AKT, PKC, and SGK [11, 12]. In diabetic models of kidney disease, nephrilin, a peptide inhibitor of mTORC2, partially protects against kidney dysfunction. In these models, it has been suggested that its action involves the disruption of a stress-induced Rictor::IRS complex [13].

In this study, the inflammatory process triggered by stress was assessed using a series of inflammatory markers of macrophage infiltration, neurogenic inflammation, and mitochondrial oxidative metabolism. The involvement of substance P-positive C-fibers in the initiation of inflammatory processes in response to traumatic stress has been previously suggested. Ubiquitin carboxy-terminal hydrolase L1 (UCHL1, PGP9.5), abundantly expressed in neurons, is a known co-regulator of anti-inflammatory p53 activity and NF-kappa-B activation [18–23]. Macrophage-mediated tissue inflammation [24] can be accompanied by disruptions in mitochondrial oxidative metabolism [25] and cell morphology, particularly the adoption of mesenchymal characteristics by epithelial cells [26]. Mitochondrial generation of reactive oxygen species is mediated by the adaptor protein p66shc [27, 28], which is itself activated by phosphorylation at serine 36 by PKC-beta-II [29].

Post-traumatic elevation in plasma inflammatory markers, such as high-mobility group protein B1 (HMGB1) and tumor necrosis factor-alpha (TNF-alpha), occurs in parallel with tissue infiltration by activated M1 macrophages and other leukocytes [14, 15], but in burn models of bacterial sepsis, for example, the exaggerated inflammatory response is not always effective at combating infection [16, 17].

In this work, we study the effect of inhibiting Rictor binding to its cofactor Protor [13, 30] on inflammatory processes triggered by xenobiotic stress in three previously described CD1 mouse models of acute xenobiotic injury [31, 32]. We also explore longer-term effects on oxidative metabolism using a salt-sensitive hypertensive Dahl rat model [33]. The major aim of this study was to define the role of mTORC2 as a transducer of cellular stress in tissue.

## Materials and methods

### Reagents

Nephrilin peptide was custom synthesized by Genemed Synthesis (San Antonio, TX, USA). Phosphosafe cell extract reagent was from Novagen (Madison, WI, USA) and BCA Protein Kit from Pierce (Rockford, IL, USA). Antibodies for ELISAs were purchased from Santa Cruz Biotechnology (Santa Cruz, CA, USA) except for phospho-p66shc-S36, phospho-PKC-alpha-T638, and phospho-beta2-T641 (Abcam, Cambridge, MA, USA). Sandwich ELISA kits for substance P, IL-6, TNF-alpha, and NGAL (lipocalin-2) were purchased from R&D Systems (Minneapolis, MN, USA). HMGB1 sandwich ELISA kit was obtained from USCN Life Science (Wuhan, China). Urine albumin and creatinine were measured using the Albuwell M Kit and Creatinine Companion Kit from Exocell (Philadelphia, PA, USA). CelLytic M cell lysis reagent, NXTRACT cell fractionation kit, LPS, glycerol, gentamycin, and cisplatin were obtained from Sigma (St. Louis, MO, USA). Quantisure First Strand cDNA Synthesis Kit (Accugen Biosciences, Rockville, MD, USA) was used for quantitative PCR (qPCR) studies.

### Animal studies

All experiments were approved to be conducted as outlined here by the appropriate institutional IACUUC animal ethics committee and carried out in accordance with established Guiding Principles for Animal Research. Male Dahl/ss rats, 6–7 weeks old (Charles River), were fed AIN76A w/0.3 % Na chow upon arrival at the facility, then either switched to AIN76A w/8 % Na (high-salt diet group) or maintained on original diet (low-salt diet group) starting on day 1. Dahl rats on high-salt diet were either left untreated or injected daily with either saline or 4 mg/kg nephrilin by subcutaneous bolus (injection volume 0.4 ml/rat) for 4 weeks. Rats were placed in metabolic cages following the final dose for a 12-h acclimation time, then a 12-h urine collection time. Acute kidney injury (AKI) models using intramuscular 50 % glycerol to induce rhabdomyolysis, intraperitoneal gentamycin (80 mg/kg), or intraperitoneal cisplatin insults were carried out essentially as previously described [31, 32], except that the dosing of gentamycin and cisplatin were done as follows: gentamycin 80 mg/kg injected twice daily (days 4 through 6), cisplatin 2.7 mg/kg daily for 5 days (days 1–5) to more closely parallel clinical practice. Seven-week-old CD-1 mice (~30 g body weight each) were obtained from Charles River Laboratories. Mice were placed in metabolic cages (5 mice per cage) for a 12-h acclimation time preceding a 12-h urine collection before sacrifice. All mice except for the sham group received daily subcutaneous injection of either saline or 4 mg/kg nephrilin, as previously described [13]. The injection volume was 0.1 ml/mouse. LPS was administered at 2 mg/kg by subcutaneous injection 18 h after the last dose of glycerol, gentamycin, or cisplatin. Blood was collected by retro-orbital bleed under isoflurane anesthesia prior to LPS administration. Animals were sacrificed 2 h later. In all Dahl rat and CD1 mouse experiments, blood was additionally collected at sacrifice by terminal cardiac puncture under CO_2_ anesthesia and processed for plasma with K-EDTA preservation at −80 °C. Left kidneys were snap frozen and stored at −80 °C for analysis. Tissues were extracted and assayed by ELISA as previously described [13]. Segments of frozen organ tissue were used for immunohistochemistry studies and for RNA extraction and OneArray™ gene array analysis as specified by the manufacturer (Phalanx Biotech, Palo Alto, CA, USA). The Accession numbers for UCHL1 and PER2 genes are NM_011670.2 and NM_011066.3, respectively.

### Cell extracts, fractionation, ELISAs and immunoprecipitation

Kidney tissue cell extracts, cell fractions and ELISAs were done as previously described [13, 34] except that the Sigma NXTRACT kit was used for cell fractionation. For immunoprecipitation of proteins bound to IRS2, kidney slices from each treatment group were homogenized in CelLytic M, diluted in PBS and immunoprecipitated in Eppendorf tubes overnight at 8 °C in 0.6 ml reaction volume containing approximately 1.5 mg total protein and 30 ug of anti-IRS2 antibody conjugated to agarose. Conjugates were pelleted by 30 s microfuge spin, and pellets were washed once in ice-cold PBS before elution in 200 uL 0.1 M Glycine. After centrifugation, eluates were immediately neutralized with 0.1 ml Trizma pH 7.8. The elution step was repeated and the eluates were pooled for each sample. All immunoprecipitation experiments were performed in duplicate and eluates were assayed in triplicate.

### Statistical analysis

Probability values (*P* values) were computed using Student’s *t* test and expressed relative to saline-treated controls, except where otherwise noted. Group size for all treatment groups was five animals except for CD1 sham group, which consisted of three animals.

### Immunohistochemistry

Frozen sections were prepared and stained with anti-phospho-p66shc-S36 antibody (Abcam 6E10, isotype IgG1, stock concentration 0.1 mg/ml). A series of IHC pilot sequences was first performed to find the correct staining protocol for the antibody. Optimal staining was found using a flash frozen rat kidney section fixed in an acetone/ethanol solution for 5 min at room temperature (RT). Prior to antibody staining, a mouse IgG block (Biocare Medical RBM961H) was applied to the tissue for 30 min at RT. The primary antibody working concentration was best at 0.002 mg/ml or (1:50) and incubated at 4 °C for 18 h. The isotype negative control solution used was Mouse IgG1 (Dako X0931) diluted to the same concentration as the working antibody solution (0.002 mg/ml). Following overnight incubation, the primary antibody was conjugated with an anti-mouse labeled polymer (Biocare Medical MM620H). Staining was developed with DAB+ (Dako K3468) for 5 min RT. Counter-staining was done with Automation Hematoxylin for 10 min, RT.

### Analysis of kidney tissue gene expression

RNA extraction, QC, gene array using Phalanx OneArray™ and collection of signal intensities were performed under contract by Phalanx Biotech. Each RNA sample was prepared from a pool of frozen kidney slices from the animals in each treatment group. RNA showed 260/280 nm ratios >1.95 and passed QC by gel electrophoresis and chromatography, signal capture, and analysis were performed as specified by the service provider (http://www.phalanxbiotech.com/services/services.html). The same RNA samples used for gene array experiments were used for first strand cDNA synthesis using Quantisure Kit, after removal of gDNA using gDNA-removing buffer (4×). Reverse transcriptase reaction contained 4 uL 5× RT buffer, 3 uL primer mix, 1 uL enzyme mix and 12 uL RNA (1 ug). After 15 min incubation at 42 °C, the reaction was terminated (95 °C for 3 min) and the cDNA was used in a qPCR reaction using the following primer sets (primers were from sequences conserved in rodents).

**Table:** 

Gene	Amplicon	Forward primer	Reverse primer
Per2	207	5′ CTGGTCCAGCTTCATCAACCC 3′	5′ TAGCCACTGGAGCCGCTGT 3′
Gapdh	107	5′ GGGCTCTCTGCTCCTCCCTGTT 3′	5′ ACGGCCAAATCCGTTCACACCG 3′
Uchl1	150	5′ ACCCCGAGATGCTGAACAAAGTG 3′	5′ GCTGGGCCGTGAGGGGAAAC 3′

Reactions contained 12.5 uL AccuAmp™ SYBR Green qPCR Master Mix/Low ROX (2×), 1 uL (300 nM) each primer, 2.5 uL 5×-diluted cDNA, 8 uL nuclease-free water (total reaction volume 25 uL). PCR cycling was performed in an Agilent MX3000 real-time cycler according to manufacturer’s instructions. Cycle conditions were set for 95 °C, 10 min as an initial activation step, followed by 40× 2-step cycling (95 °C, 15 s followed by 55 °C 1 min with fluorescence data collected during the second step). All experiments were done in triplicate. Delta-delta-CT method was used to calculate fold-change in gene expression using GAPDH as the reference gene.

## Results

### mTORC2 inhibition with nephrilin abrogates the abnormal inflammatory response to xenobiotic stress in three models of AKI

**Fig. 1:**
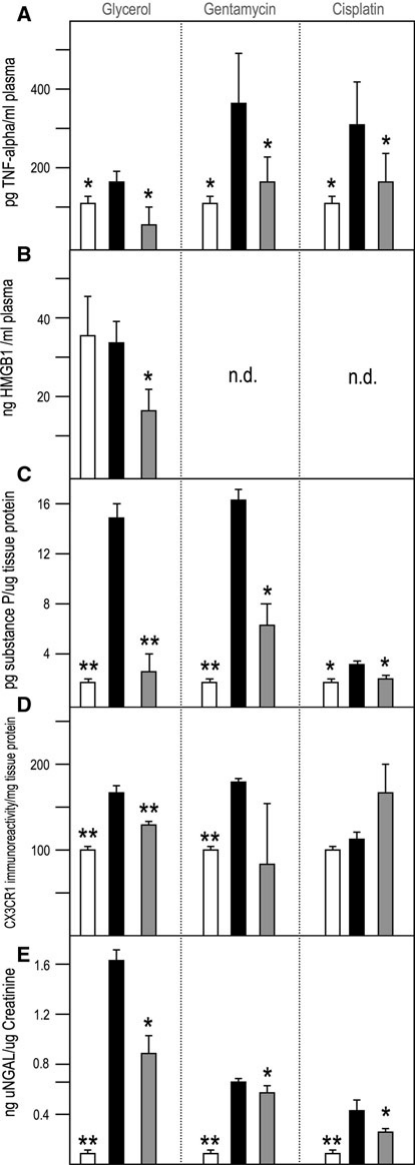
Nephrilin inhibits inflammatory responses to xenobiotic stress. Three murine models of xenobiotic insult (glycerol, gentamycin, cisplatin) were used to probe inflammatory responses to stress as described in “Materials and methods”. Group size was five animals per group. The time from initial insult to tissue analysis was 20 h, 3 and 6 days, respectively, for the three models. All samples shown were collected at sacrifice. Plasma samples taken prior to LPS challenge were confirmed to have <10 pg/ml TNF-alpha for all animals (data not shown). Pre-challenge plasma HMGB1 in rhabdomyolytic (glycerol) animals was <10 ng/ml (data not shown). **a** Plasma TNF-alpha levels. **b** Plasma HMGB1. **c** Substance P in kidney tissue extracts. **d** CX3CR1 immunoreactivity in kidney tissue extracts. **e** uNGAL-creatinine ratio in urine. No xenobiotic (*white bars*), xenobiotic (*black bars*), xenobiotic + nephrilin (*gray bars*). All animals were challenged with LPS 2 h prior to sacrifice. The same “no xenobiotic” control group data (*white bars*) are plotted for comparison with each of the three xenobiotic arms. *P < 0.05, **P < 0.01 significance relative to insult group in each experiment. *n.d.* Not determined

Acute xenobiotic or ischemic injury magnifies the inducibility of inflammatory markers in response to LPS challenge CD1 mouse models of AKI [31, 32]. We explored the effect of sub-cutaneously administered nephrilin in three of these models glycerol (rhabdomyolysis), gentamycin and cisplatin. In all three AKI models, basal levels of plasma TNF-alpha were below 10 pg/ml, but 2 h after LPS stimulation (Fig. 1a) plasma TNF-alpha levels rose to significantly higher levels in the glycerol-, gentamycin- and cisplatin-challenged mice than in control CD-1 mice (all *P* < 0.01). Treatment with nephrilin significantly reduced plasma TNF-alpha in xenobiotic-treated animals by at least 40 % in each case (*P* < 0.05).

HMGB1 is a canonical ligand for TLR4, which has been shown to mediate TNF-alpha production and kidney tissue injury in response to traumatic injury [35–37]. Plasma HMGB1 levels in the rhabdomyolysis model were significantly elevated by LPS challenge, but not further elevated by xenobiotic stress; however, elevated HMGB1 levels were significantly reduced by treatment with nephrilin (Fig. 1b).

Substance P has been implicated as an effector of neurogenic inflammation in tissues and in neuropathies [18, 19]. In kidney tissue, xenobiotic stress caused a marked elevation in substance P in all models. In each case, nephrilin significantly reduced tissue levels of substance P (Fig. 1c).

CX3CR1 is a key indicator of recruitment for M1 macrophage subsets in the context of local inflammatory responses [15]. In the glycerol and gentamycin mouse models, we observed significant insult-associated elevations in CX3CR1 immunoreactivity. In the glycerol model, treatment with nephrilin significantly lowered the level of CX3CR1 (Fig. 1d).

Urinary neutrophil gelatinase-associated lipocalin, an early marker of kidney injury [4, 5], is significantly elevated by xenobiotic stress in all three AKI models. Daily subcutaneous bolus administration of nephrilin significantly reversed this elevation in all cases (Fig. 1e).

### mTORC2 regulates the activation of p66shc in hypertensive rats

**Fig. 2:**
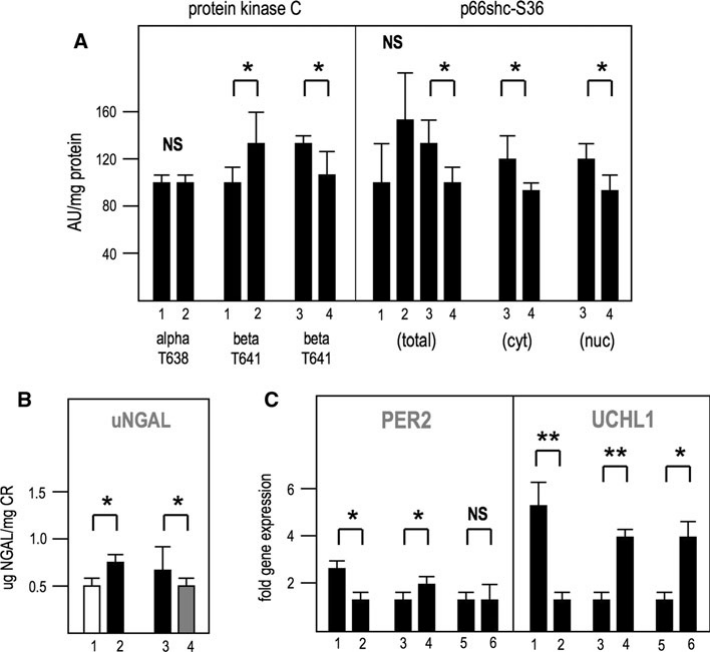
Nephrilin inhibits responses to stress. Group size was five animals per group. *NS* not significant, *P < 0.05. **a** Phosphorylation of PKC and p66shc in kidney tissue as measured by ELISA. Results are plotted in arbitrary units per mg of protein relative to the control group in each comparison (group 1 or 3). *Cyt* cytoplasmic, *Nuc* nuclear. **b** Urinary NGAL in hypertensive rats. **c** Gene expression levels relative to GAPDH, as established by qPCR. *Left* PER2 gene, *right* UCHL1 gene.* 1* Dahl rats, low-salt diet;* 2* Dahl rats, high salt diet; 3 Dahl rats, high salt + saline;* 4* Dahl rats, high salt + nephrilin;* 5* CD1 mice, glycerol treatment + saline;* 6* CD1 mice, glycerol treatment + nephrilin

**Fig. 3:**
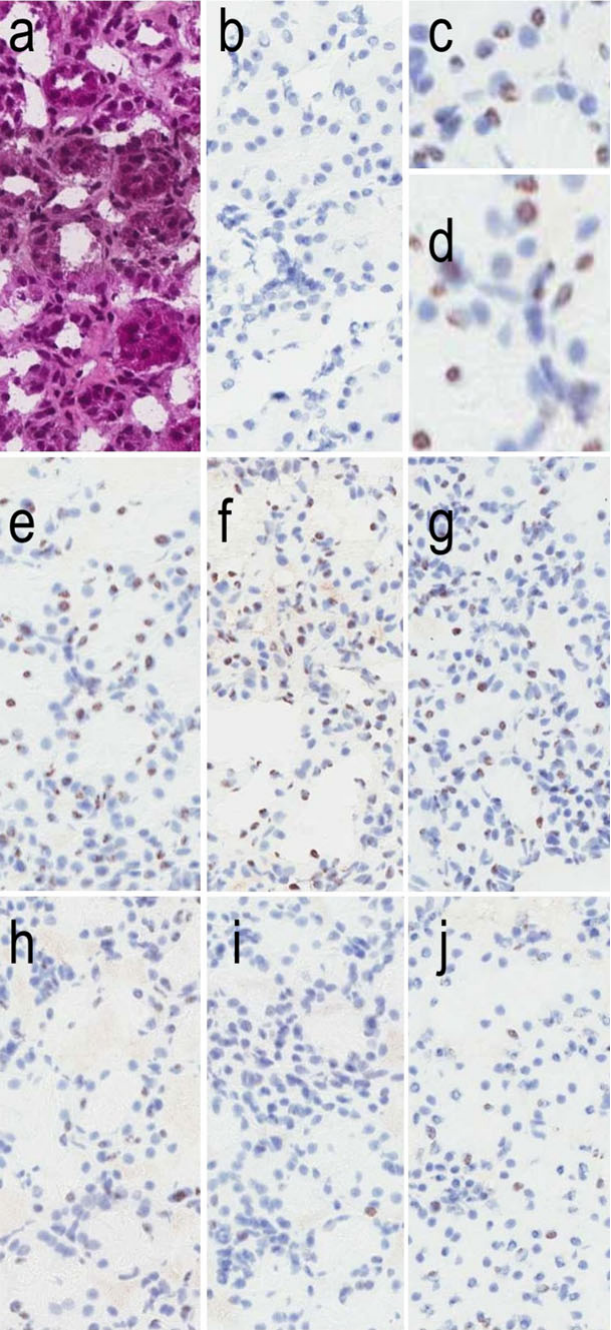
Immunohistochemical staining of frozen kidney sections with anti-phospho-p66shc-S36 antibody. **a** H&E stain, **b** isotype negative control, **c**, **d** magnification of detail in (**e**, **f**), highlighting the circular punctate nuclear staining, **e**–**g** three representative animals from saline-treated group, **h**–**j** three representative animals from nephrilin-treated group

We next investigated the impact of mTORC2 inhibition in the context of metabolic stress and oxidative metabolism. Phosphorylation of S36 in p66shc by PKC-beta-2 has been implicated as the key step in the generation of mitochondrial reactive oxygen species [29]. We have recently shown that hyperglycemic stress elevates the phosphorylation and intracellular localization of kidney PKC-alpha and PKC-beta-2 in an mTORC2-dependent manner, but the antibody used in that study could not distinguish between phosphorylation events at PKC-alpha-T638 and PKC-beta-2-T641 [13]. In the present study, we used reagents specific to each of these isoforms of phosphorylated PKC-alpha and beta-2 to examine, whether a high-salt diet increased phosphorylation at these sites in hypertensive Dahl rats. Figure 2a shows that hypertensive metabolic stress significantly elevates phosphorylation of PKC-beta-2-T641, but not PKC-alpha-T638. In Dahl rats fed a high-salt diet for 4 weeks, daily treatment with nephrilin protects against this elevation in phosphorylation of PKC-beta-2-T641. In these animals, both cytoplasmic and nuclear extracts of kidney tissues also showed significantly reduced proportions of p66shc phosphorylated at S36 after treatment with nephrilin (Fig. 2a, right panel). As PKC-beta-II has been implicated in the maturation of p66shc by S36 phosphorylation [29], these observations are consistent with a regulatory role for mTORC2 in this pathway. Maturation of PKC alpha/beta by mTORC2 kinase has been postulated to occur in a non-cytoplasmic compartment, possibly nuclear [13]. In frozen sections stained with an antibody specific to phospho-p66shc-S36, a ring-like pattern of nuclear staining is observed (see detail in panels c, d of Fig. 3), perhaps suggesting localization of phosphorylated p66shc within nuclear PML-containing stress bodies [38]. Nephrilin treatment reduced the number of dark-stained nuclei (Fig. 3; Table 1).

**Table 1:** Percent nuclei stained with phospho-p66shc-S36 antibody

Group	# nuclei	No stain	Light stain	Dark stain
Saline group	1,820	60.74 ± 4.68	10.12 ± 0.21	29.14 ± 4.47
Nephrilin group	2,745	76.11 ± 2.29*	12.99 ± 1.03**	10.90 ± 1.98*

Dahl rats were fed a high-salt diet for 4 weeks and simultaneously injected daily with saline or 4 mg/kg nephrilin. Frozen kidney sections were stained with anti-phospho-p66shc-S36

* *P* < 0.05, ** *P* < 0.01 vs. saline control

In order to confirm a functional effect of nephrilin treatment we measured uNGAL in these animals. Stress induced a significant elevation in uNGAL and this elevation was abrogated by treatment with nephrilin (Fig. 2b).

### mTORC2 regulates gene expression in response to stress

We examined differential gene expression using gene arrays, as described in “Materials and methods”. Gene arrays were interrogated for significant changes in array signal in triplicate. Gene tags with signal intensities below 100 (arbitrary units, common to all arrays) were ignored in the analysis. Three pairwise comparisons were performed (Table 2). For example, changes in kidney gene expression attributable to salt diet in Dahl rats was done by comparing mRNA from kidneys of animals fed low- or high-salt diets. We used an arbitrary cutoff of 20 % (upregulated or downregulated) with *P* < 0.05 for triplicate determinations. We examined changes in gene expression caused by stress and counter-regulated by nephrilin. Using gene arrays (Table 2) we searched for genes that were differentially expressed in both rat sets. We identified two genes: UCHL1, a protein abundantly expressed in neurons and previously implicated in ubiquitin metabolism and PER2, a well-known clock gene implicated in circadian function [39, 40].

**Table 2:** Pairwise comparisons using gene arrays

Model	Control	Comparator	# genetags
Hypertensive Dahl rat	Low-salt diet	High-salt diet	6,204
Hypertensive Dahl rat (high-salt diet)	Saline	Nephrilin	6,204
Rhabdomyolytic CD1 mouse	Saline	Nephrilin	26,423

mRNA was extracted from frozen kidney tissue and queried as described in “Materials and methods”. Three separate experiments are summarized in the table. In each experiment, the *Control* mRNA was compared with the *Comparator* mRNA to identify differentially expressed genes

# *Genetags* is the total number of genetags queried in each array

In a confirmatory step, mRNA extracted from kidneys was queried by qPCR. Data were expressed as a ratio of UCHL1 or PER2 gene transcript to the reference housekeeping gene GAPDH. The results of the experiment are shown in Fig. 2d. Both UCHL1 and PER2 were significantly downregulated in both acute and hypertensive models of kidney insult, but nephrilin treatment reversed downregulation of UCHL1 in both acute (glycerol) and hypertensive models, and reversed downregulation of PER2 in the Dahl rat model.

### Nephrilin reduces co-immunoprecipitation of Rictor with IRS2 in AKI model

We have previously shown that an intracellular complex of Rictor and IRS protein (IRS1 or IRS2) is rapidly formed in human kidney cells in response to glycemic stimulus [13]. We immunoprecipitated kidney tissue extracts from Dahl rats (high-salt diet, treated with either saline or nephrilin) and from CD1 mice (rhabdomyolysis model) using an anti-IRS2 antibody conjugated to agarose (see “Materials and methods”). The solubilized eluates from immunoprecipitates were assayed by ELISA. IGF1R was used as an internal calibration control, based on the known canonical role of IGF1R::IRS2 complexes in cellular signaling, and results were expressed as the ratio of Rictor to IGF1R immunoreactivity. As shown in Table 3, nephrilin significantly reduces the ratio of Rictor immunoprecipitated by anti-IRS2 in the AKI model, but not in the Dahl rat model.

**Table 3:** Ratio of Rictor to IGF1R co-immunoprecipitating with kidney IRS2

Group	Rictor::IGF1R ratio (AU)	*P* value^a^
Dahl rat/high-salt diet/saline	0.950 ± 0.132	
Dahl rat/high-salt diet/nephrilin	1.026 ± 0.115	0.310
CD1 mouse/sham	0.972 ± 0.065	0.160
CD1 mouse/rhabdomyolysis/saline	1.037 ± 0.081	
CD1 mouse/rhabdomyolysis/nephrilin	0.849 ± 0.077	0.002

Kidney tissue lysates were immunoprecipitated using agarose-conjugated anti-IRS2 antibody, as described in “Materials and methods”. Eluates were assayed for Rictor and IGF1R by ELISA. Ratio is expressed in arbitrary units. Experiments were performed twice. The results of one representative experiment are shown in which samples were immunoprecipitated in duplicate and each immunoprecipitate assayed by ELISA in triplicate

^a^Versus saline control group

## Discussion

The progression of events in acute injury models provides an opportunity for investigating the biochemical sequence that links traumatic stress with dysfunctional inflammation and other potentially self-damaging systemic responses. Early events associated with acute organ damage (as measured by uNGAL in the case of AKI) appear to follow tissue infiltration by activated monocytes and the release of inflammatory mediators [6, 15, 31, 32]. Our data demonstrate that nephrilin, a peptide inhibitor of mTORC2, blunts the elevation of TNF-alpha in plasma (Fig. 1a) and of uNGAL in urine (Fig. 1e). In kidney tissue, substance P is released in response to trauma (all three models; Fig. 1c) and CX3CR1 levels rise (two models; Fig. 1d), suggesting the possibility that neurogenic inflammation may play a role in linking stress to inflammation. Both responses are largely abrogated by treatment with the mTORC2 inhibitor, consistent with a regulatory role for mTORC2 (Fig. 1c, d).

The most notable effects of trauma among the markers we measured (levels of substance P in kidney tissue and uNGAL in urine; Fig. 1a, e) and the corresponding impact of nephrilin on these markers are observed in the rhabdomyolysis model. In this model, tissue collection for analysis is separated from the initial insult by only 20 h as compared with the gentamycin and cisplatin models, where the time elapsed from first insult to collection is 3 and 6 days, respectively. It is possible, therefore, that the most dramatic regulatory effects of mTORC2 are exerted on processes that occur early after the initial injury. HMGB1 is a canonical ligand for TLR4, which has been shown to mediate TNF-alpha production and kidney tissue injury in response to traumatic injury [35–37]. Plasma HMGB1 levels in the rhabdomyolysis model were significantly elevated by LPS challenge, but not further elevated by xenobiotic stress (Fig. 1b); however, the elevated HMGB1 levels were significantly reduced by treatment with nephrilin.

It is also possible, of course, that the three types of insult used in the experiments (glycerol, gentamycin, cisplatin) are just intrinsically different, and produce different responses. Future studies focused on the time course of trauma response will be needed to distinguish between these possibilities.

mTORC2 may indirectly regulate inflammation in a general sense, via its role in the maturation of AGC kinases, but we also note the possibility that it may mediate a hyper-inflammatory response to stress in animals subjected to xenobiotic insult, at least in the three AKI models used in this study. In that scenario, a neurogenic mechanism for regulation is suggested by the effects of nephrilin on substance P levels (Fig. 1c) and UCHL1 gene expression (Fig. 2d) in kidney tissue.

The potential clinical significance of these data in a critical care context is worth noting. Over the past four decades, mortality in critical care settings, particularly when associated with systemic hyper-inflammation has remained alarmingly high often exceeding 20 % [3, 41]. Recent studies have shown a strong association of such mortality with kidney damage, and with elevated uNGAL in particular [4, 5]. It should be noted, however, that these are correlational observations and do not prove a causal relationship. In some high-risk patient populations such as those undergoing major heart surgery or those with severe burns, mortality correlates with systemic inflammation and kidney insult [42, 43].

Our data also demonstrate the influence of mTORC2 on oxidative processes known to be associated with kidney injury over an extended period following the initial insult. In hypertensive Dahl rats, nephrilin significantly reduces activation of PKC-beta-II by phosphorylation at T641. PKC-beta-II has previously been shown to activate p66shc, a key mediator of mitochondrial oxidative metabolism, by phosphorylation of S36 [29]. In Dahl rats, nephrilin inhibits S36 phosphorylation of kidney p66shc. The latter process may be localized in PML-containing nuclear bodies [38], based on the ring-like pattern of immunohistochemical staining of frozen kidney sections with anti-phospho-p66shc-S36 antibody.

UCHL1 gene expression, which is subject to epigenetic silencing [44], is downregulated in rhabdomyolytic mice and in hypertensive Dahl rats fed a high-salt diet. Nephrilin treatment reverses this effect. The observation is intriguing in light of recent reports linking UCHL1 to p53 function and of both these proteins to inflammatory processes [20–23]. The UCHL1 gene is also abundantly expressed in neurons. UCHL1 gene silencing and the release of substance P in kidney tissue in response to stress may presage a change of function in nociceptors and is consistent with trauma-induced alterations in neurogenic signaling [18, 19]. In the Dahl rat model, nephrilin reverses the reduction in PER2 gene expression, a hallmark of circadian dysfunction [40].

Given the immunohistochemical staining of frozen kidney sections with anti-phospho-p66shc-S36, and the ubiquitous linkage between oxidative and inflammatory circuitry in mammalian cells, it is interesting to note that PML-containing nuclear bodies have recently been shown to contain p53 [45]. In future studies, it would be interesting to investigate the possible role of nuclear stress bodies in the coordination of p66shc- and NF-kappa-B-driven processes, and the possible involvement of UCHL1 and p53 in such putative crosstalk.

We suggest that inflammatory and oxidative circuits may be coordinately induced by stress via a temporal sequence involving (1) reduced UCHL1 gene expression (possibly by epigenetic silencing), (2) release of substance P in tissue, (3) elevation of inflammatory markers, (4) damage to kidney tubular epithelial cell function (as measured by uNGAL), and (5) activation of PKC-beta-2, which in turn activates p66shc, leading to mitochondrial ROS generation [29].

Rhabdomyolytic stress, like glycemic stress [13], appears to drive formation of an IRS2::Rictor complex in cells, and the formation of complex is abrogated by nephrilin. The role of the IRS::mTORC2 complex in mediating the inflammatory and oxidative phenomena listed above is not known. Further work is necessary to elucidate the pathways influenced by this complex.

Taken together, the above data suggest a protective effect for an inhibitor of mTORC2 in both acute xenobiotic and longer-term metabolic stress. It is tempting to speculate that, since many of the tissue processes explored in this study (inflammation, oxidative damage, circadian dysfunction) appear to underlie other degenerative diseases and aging [46–48], mTORC2 may play a broad role in degenerative mechanisms associated with those disease states. Even if mTORC2 inhibition does not address the primary causes of such diseases, it may help manage the cascade of degenerative effects set in motion by the primary insults. The generality of mTORC2 action may signal evolutionary conservation and may represent an example of antagonistic pleiotropy, a phenomenon that has recently been described in connection with regulators of inflammatory processes such as p53 and IL-6 [49, 50].

In conclusion, we have shown that inhibition of mTORC2 function using nephrilin inhibits markers of inflammatory and oxidative metabolism in rodent models featuring short-term xenobiotic or longer-term metabolic insult. These results may suggest a conserved role for mTORC2 complex in the biological response to stress.

## References

[1] Morgan MJ, Liu ZG (2011). Crosstalk of reactive oxygen species and NF-κB signaling. Cell Res.

[2] Rath E, Haller D (2011). Inflammation and cellular stress: a mechanistic link between immune-mediated and metabolically driven pathologies. Eur J Nutr.

[3] Bagshaw SM, Wald R (2010). Acute kidney injury in 2010: advances in diagnosis and estimating disease prognosis. Nat Rev Nephrol.

[4] Haase M, Bellomo R, Haase-Fielitz A (2010). Neutrophil gelatinase-associated lipocalin. Curr Opin Crit Care.

[5] Ricci Z, Cruz D, Ronco C (2008). The RIFLE criteria and mortality in acute kidney injury: a systematic review. Kidney Int.

[6] Akcay A, Nguyen Q, Edelstein CL (2009). Mediators of inflammation in acute kidney injury. Mediators Inflamm.

[7] Pi J, Zhang Q, Fu J, Woods CG, Hou Y, Corkey BE, Collins S, Andersen ME (2010). ROS signaling, oxidative stress and Nrf2 in pancreatic beta-cell function. Toxicol Appl Pharmacol.

[8] Kamp DW, Shacter E, Weitzman SA (2011). Chronic inflammation and cancer: the role of the mitochondria. Oncology.

[9] Hall MN (2008). mTOR—what does it do?. Transplant Proc.

[10] Jacinto E, Loewith R, Schmidt A, Lin S, Rüegg MA, Hall A, Hall MN (2004). Mammalian TOR complex 2 controls the actin cytoskeleton and is rapamycin insensitive. Nat Cell Biol.

[11] Facchinetti V, Ouyang W, Wei H, Soto N, Lazorchak A, Gould C, Lowry C, Newton AC, Mao Y, Miao RQ, Sessa WC, Qin J, Zhang P, Su B, Jacinto E (2008). The mammalian target of rapamycin complex 2 controls folding and stability of Akt and protein kinase C. EMBO J.

[12] García-Martínez JM, Alessi DR (2008). mTOR complex 2 (mTORC2) controls hydrophobic motif phosphorylation and activation of serum- and glucocorticoid-induced protein kinase 1 (SGK1). Biochem J.

[13] Singh BK, Singh A, Mascarenhas D (2010). A nuclear complex of Rictor and IRS2 is associated with albuminuria in diabetic mice. Metab Syn Relat Disord.

[14] Furuichi K, Kaneko S, Wada T (2009). Chemokine/chemokine receptor-mediated inflammation regulates pathologic changes from acute kidney injury to chronic kidney disease. Clin Exp Nephrol.

[15] Li L, Okusa MD (2010). Macrophages, dendritic cells, and kidney ischemia-reperfusion injury. Semin Nephrol.

[16] Asai A, Tsuda Y, Kobayashi M, Hanafusa T, Herndon DN, Suzuki F (2010). Pathogenic role of macrophages in intradermal infection of methicillin-resistant *Staphylococcus aureus* in thermally injured mice. Infect Immun.

[17] Shigematsu K, Asai A, Kobayashi M, Herndon DN, Suzuki F (2009). Enterococcus faecalis translocation in mice with severe burn injury: a pathogenic role of CCL2 and alternatively activated macrophages (M2aMphi and M2cMphi). J Leukoc Biol.

[18] Donkin JJ, Turner RJ, Hassan I, Vink R (2007). Substance P in traumatic brain injury. Prog Brain Res.

[19] Myers RR, Campana WM, Shubayev VI (2006). The role of neuroinflammation in neuropathic pain: mechanisms and therapeutic targets. Drug Discov Today.

[20] Dijsselbloem N, Goriely S, Albarani V, Gerlo S, Francoz S, Marine JC, Goldman M, Haegeman G, Vanden Berghe W (2007). A critical role for p53 in the control of NF-kappaB-dependent gene expression in TLR4-stimulated dendritic cells exposed to genistein. J Immunol.

[21] Yamanishi Y, Boyle DL, Pinkoski MJ, Mahboubi A, Lin T, Han Z, Zvaifler NJ, Green DR, Firestein GS (2002). Regulation of joint destruction and inflammation by p53 in collagen-induced arthritis. Am J Pathol.

[22] Takami Y, Nakagami H, Morishita R, Katsuya T, Cui TX, Ichikawa T, Saito Y, Hayashi H, Kikuchi Y, Nishikawa T, Baba Y, Yasuda O, Rakugi H, Ogihara T, Kaneda Y (2007). Ubiquitin carboxyl-terminal hydrolase L1, a novel deubiquitinating enzyme in the vasculature, attenuates NF-kappaB activation. Arterioscler Thromb Vasc Biol.

[23] Li L, Tao Q, Jin H, van Hasselt A, Poon FF, Wang X, Zeng MS, Jia WH, Zeng YX, Chan AT, Cao Y (2010). The tumor suppressor UCHL1 forms a complex with p53/MDM2/ARF to promote p53 signaling and is frequently silenced in nasopharyngeal carcinoma. Clin Cancer Res.

[24] Ricardo SD, van Goor H, Eddy AA (2008). Macrophage diversity in renal injury and repair. J Clin Invest.

[25] Naik E, Dixit VM (2011). Mitochondrial reactive oxygen species drive proinflammatory cytokine production. J Exp Med.

[26] Huang WY, Li ZG, Rus H, Wang X, Jose PA, Chen SY (2009). RGC-32 mediates transforming growth factor-beta-induced epithelial-mesenchymal transition in human renal proximal tubular cells. J Biol Chem.

[27] Arany I, Faisal A, Clark JS, Vera T, Baliga R, Nagamine Y (2010). p66shc-mediated mitochondrial dysfunction in renal proximal tubule cells during oxidative injury. Am J Physiol Renal Physiol.

[28] Sun L, Xiao L, Nie J, Liu FY, Ling GH, Zhu XJ, Tang WB, Chen WC, Xia YC, Zhan M, Ma MM, Peng YM, Liu H, Liu YH, Kanwar YS (2010). p66Shc mediates high-glucose and angiotensin II-induced oxidative stress renal tubular injury via mitochondrial-dependent apoptotic pathway. Am J Physiol Renal Physiol.

[29] Pinton P, Rimessi A, Marchi S, Orsini F, Migliaccio E, Giorgio M, Contursi C, Minucci S, Mantovani F, Wieckowski MR, Del Sal G, Pelicci PG, Rizzuto R (2007). Protein kinase C beta and prolyl isomerase 1 regulate mitochondrial effects of the life-span determinant p66Shc. Science.

[30] Pearce LR, Huang X, Boudeau J, Pawłowski R, Wullschleger S, Deak M, Ibrahim AF, Gourlay R, Magnuson MA, Alessi DR (2007). Identification of protor as a novel Rictor-binding component of mTOR complex-2. Biochem J.

[31] Zager RA, Johnson AC, Lund S, Hanson S (2006). Acute renal failure: determinants and characteristics of the injury-induced hyperinflammatory response. Am J Physiol Renal Physiol.

[32] Zager RA (2007). “Subclinical” gentamicin nephrotoxicity: a potential risk factor for exaggerated endotoxin-driven TNF-alpha production. Am J Physiol Renal Physiol.

[33] Koop K, Eikmans M, Wehland M, Baelde H, Ijpelaar D, Kreutz R, Kawachi H, Kerjaschki D, de Heer E, Bruijn JA (2008). Selective loss of podoplanin protein expression accompanies proteinuria and precedes alterations in podocyte morphology in a spontaneous proteinuric rat model. Am J Pathol.

[34] Singh BK, Mascarenhas DD (2008). Bioactive peptides control receptor for advanced glycated end product-induced elevation of kidney insulin receptor substrate 2 and reduce albuminuria in diabetic mice. Am J Nephrol.

[35] Cohen MJ, Brohi K, Calfee CS, Rahn P, Chesebro BB, Christiaans SC, Carles M, Howard M, Pittet JF (2009). Early release of high mobility group box nuclear protein 1 after severe trauma in humans: role of injury severity and tissue hypoperfusion. Crit Care.

[36] Klune JR, Dhupar R, Cardinal J, Billiar TR, Tsung A (2008). HMGB1: endogenous danger signaling. Mol Med.

[37] Wu H, Chen G, Wyburn KR, Yin J, Bertolino P, Eris JM, Alexander SI, Sharland AF, Chadban SJ (2007). TLR4 activation mediates kidney ischemia/reperfusion injury. J Clin Invest.

[38] Lang M, Jegou T, Chung I, Richter K, Münch S, Udvarhelyi A, Cremer C, Hemmerich P, Engelhardt J, Hell SW, Rippe K (2010). Three-dimensional organization of promyelocytic leukemia nuclear bodies. J Cell Sci.

[39] Day IN, Thompson RJ (2010). UCHL1 (PGP 9.5): neuronal biomarker and ubiquitin system protein. Prog Neurobiol.

[40] Albrecht U, Bordon A, Schmutz I, Ripperger J (2007). The multiple facets of Per2. Cold Spring Harb Symp Quant Biol.

[41] Lafrance JP, Djurdjev O, Levin A (2010). Incidence and outcomes of acute kidney injury in a referred chronic kidney disease cohort. Nephrol Dial Transplant.

[42] Christen S, Finckh B, Lykkesfeldt J, Gessler P, Frese-Schaper M, Nielsen P, Schmid ER, Schmitt B (2005). Oxidative stress precedes peak systemic inflammatory response in pediatric patients undergoing cardiopulmonary bypass operation. Free Radic Biol Med.

[43] Mosier MJ, Pham TN, Klein MB, Gibran NS, Arnoldo BD, Gamelli RL, Tompkins RG, Herndon DN (2010). Early acute kidney injury predicts progressive renal dysfunction and higher mortality in severely burned adults. J Burn Care Res.

[44] Yu J, Tao Q, Cheung KF, Jin H, Poon FF, Wang X, Li H, Cheng YY, Röcken C, Ebert MP, Chan AT, Sung JJ (2008). Epigenetic identification of ubiquitin carboxyl-terminal hydrolase L1 as a functional tumor suppressor and biomarker for hepatocellular carcinoma and other digestive tumors. Hepatology.

[45] Shen H, Maki CG (2010). p53 and p21(Waf1) are recruited to distinct PML-containing nuclear foci in irradiated and Nutlin-3a-treated U2OS cells. J Cell Biochem.

[46] Sarkar FH, Li Y, Wang Z, Kong D (2008). NF-kappaB signaling pathway and its therapeutic implications in human diseases. Int Rev Immunol.

[47] Frostegård J (2008). Systemic lupus erythematosus and cardiovascular disease. Lupus.

[48] Yang IA, Relan V, Wright CM, Davidson MR, Sriram KB, Savarimuthu Francis SM, Clarke BE, Duhig EE, Bowman RV, Fong KM (2011). Common pathogenic mechanisms and pathways in the development of COPD and lung cancer. Expert Opin Ther Targets.

[49] Cole SW, Arevalo JM, Manu K, Telzer EH, Kiang L, Bower JE, Irwin MR, Fuligni AJ (2011). Antagonistic pleiotropy at the human IL6 promoter confers genetic resilience to the pro-inflammatory effects of adverse social conditions in adolescence. Dev Psychol.

[50] Ungewitter E, Scrable H (2009). Antagonistic pleiotropy and p53. Mech Ageing Dev.

